# A meta-analysis and real-world cohort study on the sex-related differences in efficacy and safety of immunotherapy for hepatocellular carcinoma

**DOI:** 10.1016/j.jhepr.2023.100982

**Published:** 2023-12-12

**Authors:** Lorenz Balcar, Bernhard Scheiner, Claudia Angela Maria Fulgenzi, Antonio D’Alessio, Katharina Pomej, Marta Bofill Roig, Elias Laurin Meyer, Jaekyung Che, Naoshi Nishida, Pei-Chang Lee, Linda Wu, Celina Ang, Anja Krall, Anwaar Saeed, Bernardo Stefanini, Antonella Cammarota, Tiziana Pressiani, Yehia I. Abugabal, Shadi Chamseddine, Brooke Wietharn, Alessandro Parisi, Yi-Hsiang Huang, Samuel Phen, Caterina Vivaldi, Francesca Salani, Gianluca Masi, Dominik Bettinger, Arndt Vogel, Johann von Felden, Kornelius Schulze, Marianna Silletta, Michael Trauner, Adel Samson, Henning Wege, Fabio Piscaglia, Peter R. Galle, Rudolf Stauber, Masatoshi Kudo, Amit G. Singal, Aleena Itani, Susanna V. Ulahannan, Neehar D. Parikh, Alessio Cortellini, Ahmed Kaseb, Lorenza Rimassa, Hong Jae Chon, David J. Pinato, Matthias Pinter

**Affiliations:** 1 Division of Gastroenterology and Hepatology, Department of Internal Medicine III, Medical University of Vienna, Vienna, Austria; 2 Liver Cancer (HCC) Study Group Vienna, Division of Gastroenterology and Hepatology, Department of Internal Medicine III, Medical University of Vienna, Vienna, Austria; 3 Division of Cancer, Department of Surgery and Cancer, Imperial College London, London, United Kingdom; 4 Operative Research Unit of Medical Oncology, Fondazione Policlinico Universitario Campus Bio-Medico, Via Alvaro del Portillo, 200 - 00128 Roma, Italy; 5 Division of Oncology, Department of Translational Medicine, University of Piemonte Orientale, Novara, Italy; 6 Section for Medical Statistics, Center for Medical Data Science, Medical University of Vienna, Vienna, Austria; 7 Berry Consultants, Vienna, Austria; 8 Medical Oncology, Department of Internal Medicine, CHA Bundang Medical Centre, CHA University, Seongnam, Republic of Korea; 9 Department of Gastroenterology and Hepatology, Kindai University, Faculty of Medicine, Osaka, Japan; 10 Division of Gastroenterology and Hepatology, Department of Medicine, Taipei Veterans General Hospital, Taipei, Taiwan; 11 Department of Medicine, Division of Hematology/Oncology, Tisch Cancer Institute, Mount Sinai Hospital, New York, NY, USA; 12 Division of Gastroenterology and Hepatology, Department of Internal Medicine, Medical University of Graz, Graz, Austria; 13 Division of Hematology/Oncology, Department of Medicine, University of Pittsburgh (UPMC), Pittsburgh, PA, USA; 14 Division of Internal Medicine, Hepatobiliary and Immunoallergic Diseases, IRCCS Azienda Ospedaliero-Universitaria di Bologna, Bologna, Italy; 15 Drug Development Unit, Sarah Cannon Research Institute UK, London, UK; 16 Department of Biomedical Sciences, Humanitas University, Pieve Emanuele (Milan), Italy; 17 Medical Oncology and Hematology Unit, Humanitas Cancer Center, IRCCS Humanitas Research Hospital, Rozzano (Milan), Italy; 18 Dept of Gastrointestinal Medical Oncology, The University of Texas MD Anderson Cancer Center, Houston, TX, USA; 19 Division of Medical Oncology, Department of Medicine, University of Kansas Cancer Center, Westwood, KS, USA; 20 Department of Oncology, Università Politecnica delle Marche, Azienda Ospedaliero-Universitaria delle Marche, Ancona, Italy; 21 Institute of Clinical Medicine, National Yang Ming Chiao Tung University School of Medicine, Taipei, Taiwan; Healthcare and Services Center, Division of Gastroenterology and Hepatology, Taipei Veterans General Hospital, Taipei, Taiwan; 22 Department of Internal Medicine, University of Texas Southwestern Medical Center, Dallas, TX, USA; 23 Unit of Medical Oncology 2, University Hospital of Pisa, Pisa, Italy; 24 Department of Translational Research and New Technologies in Medicine and Surgery, University of Pisa, Pisa, Italy; 25 Department of Medicine II (Gastroenterology, Hepatology, Endocrinology, and Infectious Diseases), Freiburg University Medical Centre, Freiburg, Germany; 26 Department of Gastroenterology, Hepatology and Endocrinology, Hannover Medical School, Hannover, Germany; 27 I. Department of Medicine, University Medical Center Hamburg-Eppendorf, Hamburg, Hamburg, Germany; 28 Leeds Institute of Medical Research at St. James's (LIMR), School of Medicine, Faculty of Medicine and Health, University of Leeds, St James's University Hospital, Leeds, UK; 29 I. Medical Department, University Medical Centre Mainz, Mainz, Germany; 30 Stephenson Cancer Center, University of Oklahoma Health Sciences Center, Oklahoma City, OK, USA; 31 Department of Internal Medicine, University of Michigan, Ann Arbor, Michigan, USA

**Keywords:** gender, liver cancer, gender medicine, immunotherapy, sex

## Abstract

**Background & Aims:**

Sex-related differences in the immune pathogenesis of hepatocellular carcinoma (HCC), particularly related to oestrogen-dependent secretion of pro-tumourigenic cytokines, are well-known. Whether sex influences the efficacy and safety of immunotherapy is not known.

**Methods:**

We performed a restricted maximum likelihood random effects meta-analysis of five phase III trials that evaluated immune checkpoint inhibitors (ICIs) in advanced HCC and reported overall survival (OS) hazard ratios (HRs) stratified by sex to evaluate sex-related differences in OS. In a real-world cohort of 840 patients with HCC from 22 centres included between 2018 and 2023, we directly compared the efficacy and safety of atezolizumab + bevacizumab (A+B) between sexes. Radiological response was reported according to RECIST v1.1. Uni- and multivariable Cox regression analyses were performed for OS and progression-free survival (PFS).

**Results:**

In the meta-analysis, immunotherapy was associated with a significant OS benefit only in male (pooled HR 0.79; 95% CI 0.73–0.86) but not in female (pooled HR 0.85; 95% CI 0.70–1.03) patients with HCC. When directly comparing model estimates, no differences in the treatment effect between sexes were observed. Among 840 patients, 677 (81%) were male (mean age 66 ± 11 years), and 163 (19%) were female (mean age 67 ± 12 years). Type and severity of adverse events were similar between the two groups. OS and PFS were comparable between males and females upon uni- and multivariable analyses (aHR for OS and PFS: 0.79, 95% CI 0.59–1.04; 1.02, 95% CI 0.80–1.30, respectively). Objective response rates (24%/22%) and disease control rates (59%/59%) were also similar between sexes.

**Conclusion:**

Female phase III trial participants experienced smaller OS benefit following ICI therapy for advanced HCC, while outcomes following A+B treatment were comparable between sexes in a large real-world database. Based on the ambiguous sex-related differences in survival observed here, further investigation of sex-specific clinical and biologic determinants of responsiveness and survival following ICIs are warranted.

**Impact and implications:**

While immune checkpoint inhibitors have emerged as standard of care for the treatment of hepatocellular carcinoma, there are conflicting reports on whether the efficacy of cancer immunotherapy differs between females and males. Our study suggests ambiguous sex-related differences in outcomes from immunotherapy in hepatocellular carcinoma. Further investigation of sex-specific clustering in clinicopathologic and immunologic determinants of responsiveness to immune checkpoint inhibitor therapy should be prioritised.

**Systematic review registration:**

PROSPERO CRD42023429625.

## Highlights

• There are conflicting reports on the efficacy of cancer immunotherapy between sexes.
• Female phase III trial patients experienced worse OS following ICI therapy for HCC.
• Our study suggests ambiguous sex differences in terms of outcomes after ICI therapy in HCC.
• Further investigation of sex-specific clustering should be prioritised.

## Graphical abstract

**Figure:**
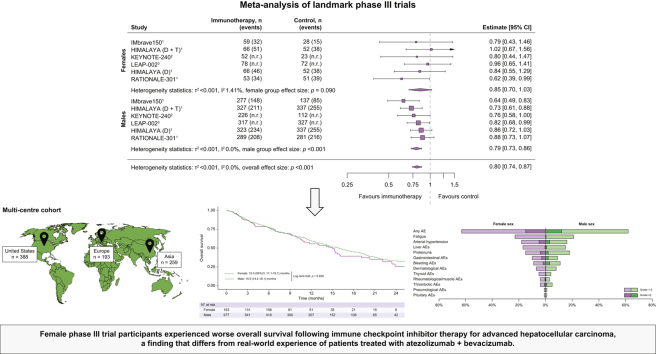

## Introduction

Advanced-stage hepatocellular carcinoma (HCC) has historically been associated with a poor prognosis due to limited systemic treatment options. However, the treatment landscape has changed rapidly in recent years. After tyrosine kinase inhibitors (TKIs) dominated the field for over a decade,^1^ the recent addition of immune checkpoint inhibitor (ICI)-based combinations of atezolizumab plus bevacizumab (A+B) and tremelimumab plus durvalumab (T+D) have increased options for first-line systemic therapy.^2^^,^^3^ While ICIs represent the mainstay of treatment across a wide variety of malignancies, there are conflicting reports as to whether the outcomes from cancer immunotherapy may differ between females and males.^4^^,^^5^

There are several immunological, biological, and behavioural differences between females and males that may affect efficacy and safety of immunotherapy. Sex-related differences in the regulation of innate and adaptive immune responses are known to play a role in hepatocarcinogenesis^6^ and may also influence response to immunotherapy.^7^ According to preclinical studies, sex hormone-associated differences such as oestrogen-mediated inhibition of IL-6 expression reduced the risk of HCC development in female animals.^6^ Sex hormones may also modulate expression and function of programmed cell death 1 (PD-1) and PD-1 ligand 1 (PD-L1), and the effects of oestrogen on PD-1 signalling play an important role in mediating autoimmunity.[8], [9], [10] It has been postulated that male patients might derive a larger relative benefit from ICI than female patients since tumours in females may be less immunogenic and enriched with more potent mechanisms of immune escape than tumours in males.^4^^,^^11^^,^^12^ In addition, there are confounding behaviours (*e.g*., smoking) which may be unequally distributed between sexes displaying strong positive co-associations with increased tumour mutational burden and ICI efficacy.^4^^,^^13^

Studies exploring the interaction between patients’ sex and the safety and efficacy of immunotherapy are scarce in patients with advanced HCC.

To fill this knowledge gap, we designed this study to systematically assess potential sex differences in overall survival (OS) in phase III clinical trials testing immunotherapy in advanced HCC. We also examined sex differences in the AB-Real study; a global, multicentre cohort of patients with HCC treated with A+B in routine clinical care.

## Patients and methods

### Meta-analysis of phase III randomised-controlled trials

We fitted a restricted maximum likelihood random effects model including all available subgroup analyses of OS data in patients with HCC stratified by sex. Inclusion criteria were: (i) phase III randomised-controlled trials (RCTs) in the palliative treatment setting, (ii) evaluation of ICIs alone or in combination with other systemic agents, (iii) OS being a primary endpoint, and (iv) available subgroup analysis of OS stratified by sex. Studies evaluating loco-regional therapies as monotherapy or in combination with systemic treatments, as well as trials evaluating systemic treatments in a (neo)adjuvant setting, were excluded.

The literature search was restricted to studies published in English and conducted in MEDLINE (https://pubmed.ncbi.nlm.nih.gov), and Embase (www.embase.com) between 1^st^ of January 2007 and 21^st^ of May 2023. Conference abstracts published until 21^st^ of May 2023 were also retrieved from the following major scientific societies: the American Society of Clinical Oncology, the European Society of Medical Oncology, the European Association for the Study of the Liver, and the American Association for the Study of Liver Diseases. The complete search strategy is reported in the Supplementary Methods 1. The study protocol was registered in PROSPERO, an international prospective register of systematic reviews (registration code CRD42023429625; https://www.crd.york.ac.uk/prospero/#searchadvanced).

We screened 11,089 studies, leading to an identification of 10 phase III trials for analysis (Fig. S1). Sex-specific OS data was available in five clinical trials. We extracted hazard ratios (HRs) for patient sex subgroups from unstratified Cox proportional-hazards models with 95% CIs for OS. The meta-analysis was calculated using the ‘metafor’ package (https://cran.r-project.org/web/packages/metafor/metafor.pdf).^14^ A funnel-plot including all different studies (Fig. S2) shows a low probability of inclusion bias.

To investigate potential differences in treatment effect between male and female patients, we calculated the differences of the log HRs in male and female patients for each phase III study, as well as the corresponding standard errors. We then performed a random-effect meta-analysis to account for potential between-study heterogeneity. A Forest plot of the random-effect meta-analysis is displayed in Fig. S3.

### The AB-Real cohort of patients with HCC treated with atezolizumab plus bevacizumab

Patients with histologically or radiologically diagnosed HCC who received A+B between May 2018 and January 2023 were included. Patients were retrospectively recruited by an international consortium including 22 centres from three different continents (Asia, Europe, and Northern America). Eligible patients were required to fulfil the following inclusion criteria: i) diagnosis of HCC by histopathological confirmation or imaging criteria according to the American Association for the Study of Liver Diseases^15^ or the European Association for the Study of the Liver^16^ guidelines, as well as ii) treatment initiation of A+B. Overall, the multicentre database included 840 eligible patients. Demographic and clinical data were collected retrospectively and curated at each participating centre. Ethical approval to conduct this study was granted by the Imperial College Institutional Review Board (Reference Number R16008).

### Reporting of sex

As suggested by guidelines on reporting of sex,^17^ the preferred terms used throughout this manuscript are sex, female and male sex. The sex of human research participants was defined based on self-reporting.^18^

### Study endpoints

This work aimed to determine differences in outcomes, treatment efficacy and safety aspects in female *vs.* male patients with HCC treated with A+B. Radiological response was evaluated by the treating physician according to RECIST v1.1 criteria. Disease control rate (DCR) was defined as the proportion of patients achieving stable disease (SD) or partial/complete response as best overall response (BOR), while objective response rate (ORR) reflected the proportion of patients with partial/complete response. The date of A+B initiation was considered as baseline for this study. Patients were followed until death or last follow-up (for censored patients) and patients alive at the data cut-off were censored at the date of the last clinical follow-up.

We aimed to document potential sex-related differences regarding baseline patient, tumour, and liver disease characteristics, and to evaluate efficacy (*i.e*., OS, progression-free survival [PFS], time to progression [TTP], BOR) as well as safety (*i.e*., adverse events [AEs]) according to sex.

Safety was reported as the incidence of AEs according to CTCAE version 4.0 or 5.0. The grading and causality of the AEs were assessed locally by the treating physicians.

### Statistical analyses

Statistical analyses were performed using R 4.3.1 (R Core Team, R Foundation for Statistical Computing, Vienna, Austria). All available patients fulfilling inclusion criteria were considered for this study. Data on baseline patient and tumour characteristics as well as radiographic features were summarised using descriptive statistics. Categorical variables were reported as absolute (n) and relative frequencies (%), while continuous variables were reported as mean ± SD or median (IQR), as appropriate. Student’s *t* test was used for group comparisons of normally distributed variables and Mann-Whitney *U* test for non-normally distributed variables. Group comparisons of categorical variables were performed using either Chi-squared or Fisher’s exact test, when the expected count in at least one cell was equal to or below 5.

OS was defined as the time from treatment initiation until death, and patients who were still alive or lost to follow-up were censored at the date of last contact. PFS was defined as time to radiological progression or death, whatever came first; patients alive or lost to follow-up without radiological progression were censored at the date of last contact. TTP was defined as time from treatment initiation until radiological tumour progression and only patients with available radiological re-staging were included in this analysis. Time on treatment was defined as the time from treatment start until end of treatment; patients who were alive or lost to follow-up with ongoing treatment were censored at the date of last contact. Median OS/PFS/TTP/time on treatment were calculated by the Kaplan-Meier method. Median estimated follow-up was calculated using the reverse Kaplan-Meier method.^19^

Univariable and multivariable analyses were conducted with Cox regression analyses. We also performed a subgroup analysis in those patients fulfilling the main inclusion criteria of the pivotal IMbrave150 phase III study (*i.e*., first-line treatment, Child-Turcotte-Pugh [CTP] stage A, Eastern Cooperative Oncology Group performance status [ECOG-PS] 0-1).^3^

The level of significance was set at a two-sided *p* value <0.05.

## Results

### Meta-analysis of phase III randomised-controlled trials

Of 10 phase III RCTs identified,^2^^,^[20], [21], [22], [23], [24], [25], [26], [27], [28] only five studies reported OS according to sex and were therefore selected for this meta-analysis: IMbrave150,^20^ HIMALAYA,^2^ KEYNOTE-240,^22^ LEAP-002,^21^ RATIONALE-301.^26^ These tested the following treatments for advanced HCC, respectively: A+B *vs.* sorafenib, D+T or durvalumab monotherapy *vs.* sorafenib, pembrolizumab *vs.* placebo, pembrolizumab plus lenvatinib *vs.* lenvatinib plus placebo, and tislelizumab *vs.* sorafenib. Overall, 5,169 patients (n = 908 female and n = 4,261 male) were included in the analysis. Sorafenib was the control arm of all the studies, except for the KEYNOTE-240 trial, which tested pembrolizumab against placebo, and the LEAP-002 trial, which used lenvatinib in the control arm. The inclusion criteria appeared to be largely consistent between trials.

**Fig. 1:**
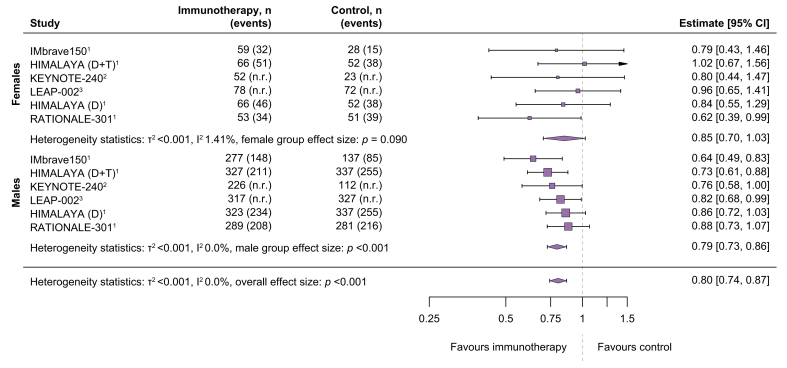
**Meta-analysis of five randomised-controlled phase III trials of immune checkpoint inhibitor-based systemic therapies for advanced hepatocellular carcinoma separated into subgroups according to sex.** A restricted maximum likelihood random effects model was used.

The OS benefit of the whole cohort (including females and males) was 20% (overall pooled HR 0.80, 95% CI 0.74–0.87; Fig. 1). A low degree of heterogeneity in the HR was indicated by I^2^ = 0% and τ^2^ = <0.001 in the overall effect size model. In pooled subgroup analyses, findings revealed a significant survival advantage among male patients (pooled HR 0.79, 95% CI 0.73–0.86). While the pooled HR for female patients was only slightly higher than the one observed in males, the OS benefit did not reach statistical significance in females (pooled HR 0.85, 95% CI 0.70–1.03; Fig. 1).

The heterogeneity test of the differences between coefficients was in favour of homogeneity between the two groups and respective studies. The estimated treatment effect difference between sexes was 0.07 (95% CI 2.02−2.16), suggesting that no differences in the treatment effect between female and male patients were observed (Fig. S3; a positive value would correspond to a greater immunotherapy effect in males compared to females).

### Study population and patient characteristics of the AB-Real cohort

Eight-hundred and forty patients were included in the second part of this study (Fig. S4). While 677 patients (81%) were male, 163 individuals (19%) were female. Mean age was 66 ± 12 years and mean BMI was 26 ± 5 kg/m^2^. The main aetiologies of liver disease were viral hepatitis (n = 380, 45%), and alcohol-related liver disease (n = 134, 16%). Most patients had established cirrhosis (n = 642, 76%). Mean CTP score was 6 ± 1 points (CTP stage A: n = 629, 75%, CTP stage B: n = 163, 19%, CTP stage C: n = 9, 1%) and mean ALBI (albumin-bilirubin) score was −2.3 ± 0.6 points. Most patients were classified as BCLC (Barcelona Clinic Liver Cancer) stage C (n = 634, 76%). Overall, 476 patients (57%) had prior surgery/local therapies, 359 individuals had extrahepatic metastases (43%), and almost half of patients had an ECOG-PS of 0 (n = 365, 44%). Ninety-five patients (11%) were receiving second or further lines of systemic treatment. Detailed patient characteristics and laboratory parameters are displayed in Table 1.

**Table 1:** Baseline patient characteristics.

	Study cohort, N = 840	Female, n = 163	Male, n = 677	*p* value
Age, years, mean ± SD	66.3 ± 11.5	67.3 ± 11.7	66.1 ± 11.4	0.240
BMI, kg/m^2^	25.6 ± 5.1	25.3 ± 5.7	25.7 ± 4.9	0.371
Cirrhosis, n (%)	642 (76%)	123 (76%)	519 (77%)	0.746
Aetiology, n (%)				
Viral	380 (45%)	82 (50%)	298 (44%)	**<0.001**
ArLD	134 (16%)	12 (7%)	122 (18%)	
ArLD/Viral	101 (12%)	28 (17%)	73 (11%)	
Other/Unknown	134 (16%)	32 (20%)	102 (15%)	
MASLD	91 (11%)	9 (6%)	82 (12%)	
CTP score, points, mean ± SD (n = 801)	6 ± 1	5.8 ± 1.0	5.9 ± 1.2	0.099
A, n (%)	629 (75%)	128 (79%)	501 (74%)	0.452
B, n (%)	163 (19%)	27 (17%)	136 (20%)	
C, n (%)	9 (1%)	1 (0.6%)	8 (1%)	
ALBI score, mean ± SD (n = 830)	−2.3 ± 0.6	−2.4 ± 0.6	−2.3 ± 0.6	0.294
Stage 1, n (%)	292 (35%)	62 (38%)	230 (34%)	0.423
Stage 2, n (%)	482 (57%)	85 (52%)	397 (59%)	
Stage 3, n (%)	56 (7%)	12 (7%)	44 (7%)	
BCLC classification, n (%) (n = 830)				
Stage A, n (%)	42 (5%)	12 (7%)	30 (4%)	0.286
Stage B, n (%)	142 (17%)	24 (15%)	118 (17%)	
Stage C, n (%)	636 (76%)	122 (75%)	514 (76%)	
Stage D, n (%)	10 (1%)	1 (0.6%)	9 (1%)	
Prior surgery/local therapy, n (%)	476 (57%)	102 (63%)	374 (55%)	0.090
Prior systemic therapy, n (%)	95 (11%)	15 (9%)	80 (12%)	0.344
Macrovascular invasion, n (%) (n = 759)	277 (33%)	45 (28%)	232 (34%)	0.294
Extrahepatic spread, n (%) (n = 806)	359 (43%)	73 (45%)	286 (42%)	0.427
ECOG-PS, n (%) (n = 823)				
0	365 (43%)	74 (45%)	291 (43%)	0.475
≥1	458 (55%)	83 (51%)	375 (55%)	
Laboratory parameters, mean ± SD or median (IQR)				
AFP, ng/dl (n = 822)	94 (8–1970)	89 (7–3703)	94 (8–1846)	0.599

Categorical variables were reported as absolute (n) and relative frequencies (%), while continuous variables were reported as mean ± SD or median (IQR), as appropriate. Student’s *t* test was used for group comparisons of normally distributed variables and Mann-Whitney *U* test for non-normally distributed variables. Group comparisons of categorical variables were performed using either Chi-squared or Fisher’s exact test, as appropriate. *P* values in bold denote statistical significance.

AFP, alpha-fetoprotein; ALBI, albumin-bilirubin; ArLD alcohol-related liver disease; BCLC, Barcelona Clinic Liver Cancer; CTP, Child-Turcotte-Pugh; ECOG-PS, Eastern Cooperative Oncology Group performance status; MASLD, metabolic dysfunction-associated steatotic liver disease.

### Differences in patient characteristics at study inclusion according to sex

Baseline characteristics, including mean age, BMI, prevalence of cirrhosis, liver function, BCLC stage, and performance status, were comparable between females and males, with only underlying aetiology being significantly different (Table 1). While the proportion of patients with viral hepatitis was higher in females (females: n = 82, 50%; males: n = 298, 44%), alcohol-related liver disease was more common in males (females: n = 12, 7%; males: n = 122, 18%; Table 1).

### Outcomes according to sex

**Fig. 2:**
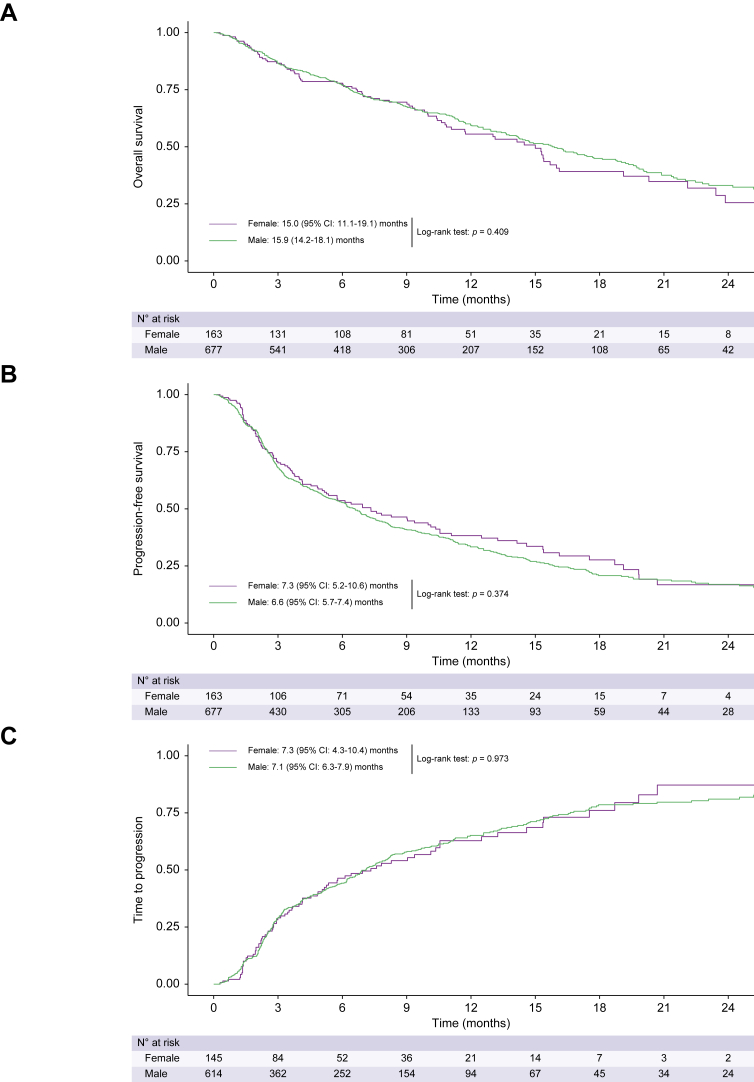
**Kaplan-Meier curves for overall survival, progression-free survival, time to progression of patients with hepatocellular carcinoma treated with atezolizumab plus bevacizumab according to sex.** (A) Overall survival, (B) progression-free survival, and (C) time to progression of patients with hepatocellular carcinoma treated with atezolizumab plus bevacizumab according to sex.

Median estimated follow-up for females and males was 14.1 (95% CI 12.4–15.9) and 12.6 (95% CI 11.1–14.2) months (*p =* 0.588; Table 2). Median OS of females was 15.0 (95% CI 11.1–19.1) months compared to 15.9 (95% CI 14.2–18.1) months for males (*p =* 0.409) (Fig. 2A, Table 2). Similar results were seen for PFS (females: 7.3 [95% CI 5.2–10.6] months *vs.* males: 6.6 [95% CI 5.7–7.4] months; *p =* 0.374; Fig. 2B, Table 2) and TTP (females: 7.3 [95% CI 4.3–10.4] months *vs.* males: 7.1 [95% CI 6.3–7.9] months; *p =* 0.973; Fig. 2C, Table 2). In univariable Cox regression analyses, sex was neither associated with OS (HR 0.90, 95% CI 0.70–1.16, *p =* 0.410), nor PFS (HR 1.10, 95% CI 0.89–1.36, *p =* 0.375) or TTP (HR 1.00, 95% CI 0.79–1.28, *p =* 0.974). This was confirmed in multivariable models including important prognostic factors (*i.e*., ascites, ALBI score, presence of extrahepatic metastases, macrovascular invasion, ECOG-PS ≥1 or alpha-fetoprotein levels ≥400 ng/dl) (Table 3). BOR, which was evaluable in 687 patients (82%), was similar between males and females. The ORR and DCR for males *vs.* females were 22% *vs.* 24% (*p =* 0.667) and 59% *vs.* 59% (*p =* 0.638), respectively (Table 2). Thirty-one percent of both females (n = 51) and males (n = 209) received a further line of systemic treatment after A+B treatment discontinuation.

**Table 2:** Efficacy outcomes according to sex.

	Study cohort, N = 840	Female, n = 163	Male, n = 677	*p* value
Median time on treatment, months (95% CI)∗	5.3 (4.6–6.0)	5.0 (3.2–6.8)	5.5 (4.7–6.3)	0.656
Median estimated follow-up, months (reverse Kaplan-Meier method) (95% CI)∗	13.3 (12.0–14.5)	14.1 (12.4–15.9)	12.6 (11.1–14.2)	0.588
Best overall response (according to RECISTv1.1), n (%)				
Not available	153 (18%)	32 (20%)	121 (18%)	0.424
Complete response	30 (4%)	2 (1%)	28 (4%)	
Partial regression	164 (20%)	33 (20%)	131 (19%)	
Stable disease	298 (36%)	61 (37%)	237 (35%)	
Progressive disease	195 (23%)	35 (22%)	160 (24%)	
Objective response rate, n (%)	194 (23%)	35 (22%)	159 (24%)	0.667
Disease control rate, n (%)	492 (59%)	96 (59%)	396 (59%)	0.638
Median overall survival, months (95% CI)∗	15.4 (13.9–16.8)	15.0 (11.1–19.1)	15.9 (14.2–18.1)	0.409
Median progression-free survival, months (95% CI)∗	6.6 (5.7–7.5)	7.3 (5.2–10.6)	6.6 (5.7–7.4)	0.374
Median time to progression, months (95% CI)∗	7.1 (6.3–7.9)	7.3 (4.3–10.4)	7.1 (6.3–7.9)	0.973

Categorical variables were reported as absolute (n) and relative frequencies (%). Group comparisons of categorical variables were performed using either Chi-squared or Fisher’s exact test, as appropriate.

∗ Compared by means of the log-rank test.

**Table 3:** Uni- and multivariable Cox regression analyses of factors associated with overall survival, progression-free survival, and time to progression.

	Univariable		Multivariable	
	HR (95% CI)	*p* value	aHR (95% CI)	*p* value
**Overall survival**				
Age, per year	1.00 (0.99–1.01)	0.794	—	—
Sex, male *vs.* female	0.90 (0.70–1.16)	0.410	0.80 (0.61–1.06)	0.114
Cirrhosis, *vs.* non-cirrhotic	1.02 (0.80–1.31)	0.866	—	—
Aetiology				
ArLD	1	—	—	—
Viral	0.96 (0.71–1.30)	0.794	—	—
MASLD	1.10 (0.74–1.65)	0.628	—	—
ArLD/Viral	1.07 (0.73–1.55)	0.739	—	—
Other/unknown	0.92 (1.08–1.34)	0.669	—	—
Presence of ascites	1.78 (1.42–2.22)	**<0.001**	1.22 (0.94–1.59)	0.131
ALBI score, per point	2.84 (2.39–3.38)	**<0.001**	2.72 (2.24–3.31)	**<0.001**
Macrovascular invasion	2.08 (1.68–2.58)	**<0.001**	1.64 (1.30–2.08)	**<0.001**
Extrahepatic spread	0.98 (0.79–1.20)	0.813	—	—
ECOG-PS ≥1, *vs.* 0	1.51 (1.23–1.86)	**<0.001**	1.18 (0.93–1.50)	0.178
AFP, ≥400 ng/dl *vs.* <400 ng/dl	1.59 (1.30–1.96)	**<0.001**	1.37 (1.09–1.72)	**0.007**
Platelets, per G/L	1.00 (0.99–1.00)	0.513	—	—
**Progression-free survival**				
Age, per year	0.99 (0.99–1.00)	0.145	—	—
Sex, male *vs.* female	1.10 (0.89–1.36)	0.375	1.04 (0.81–1.32)	0.767
Cirrhosis, *vs.* non-cirrhotic	0.94 (0.77–1.14)	0.523	—	—
Aetiology				
ArLD	1	—	—	—
Viral	1.19 (0.93–1.53)	0.165	—	—
MASLD	1.20 (0.86–1.66)	0.287	—	—
ArLD/Viral	1.07 (0.77–1.48)	0.699	—	—
Other/unknown	1.05 (0.78–1.43)	0.745	—	—
Presence of ascites	1.60 (1.32–1.93)	**<0.001**	1.19 (0.95–1.49)	0.122
ALBI score, per point	1.66 (1.44–1.91)	**<0.001**	1.46 (1.24–1.72)	**<0.001**
Macrovascular invasion	1.50 (1.25–1.79)	**<0.001**	1.26 (1.03–1.54)	**0.025**
Extrahepatic spread	1.20 (1.02–1.42)	**0.031**	1.18 (0.97–1.43)	0.089
ECOG-PS ≥1, *vs.* 0	1.45 (1.22–1.72)	**<0.001**	1.35 (1.11–1.66)	**0.003**
AFP, ≥400 ng/dl *vs.* <400 ng/dl	1.53 (1.29–1.81)	**<0.001**	1.29 (1.06–1.57)	**0.011**
Platelets, per G/L	1.00 (0.99–1.00)	0.311	—	—
**Time to progression**				
Age, per year	0.99 (0.99–1.00)	0.191	—	—
Sex, male *vs.* female	1.00 (0.79–1.28)	0.974	0.97 (0.74–1.27)	0.798
Cirrhosis, *vs.* non-cirrhotic	0.80 (0.64–0.99)	**0.041**	0.76 (0.59–0.96)	**0.025**
Aetiology				
ArLD	1	—	—	—
Viral	1.22 (0.90–1.63)	0.196	—	—
MASLD	1.27 (0.87–1.85)	0.225	—	—
ArLD/Viral	1.15 (0.79–1.68)	0.468	—	—
Other/unknown	1.17 (0.86–1.66)	0.391	—	—
Presence of ascites	1.41 (1.13–1.75)	**0.002**	1.25 (0.97–1.60)	0.084
ALBI score, per point	1.43 (1.21–1.68)	**<0.001**	1.37 (1.14–1.65)	**<0.001**
Macrovascular invasion	1.34 (1.09–1.64)	**0.006**	1.18 (0.94–1.47)	0.146
Extrahepatic spread	1.15 (0.95–1.39)	0.156	—	—
ECOG-PS ≥1, *vs.* 0	1.24 (1.02–1.49)	**0.029**	1.24 (1.00–1.55)	**0.049**
AFP, ≥400 ng/dl *vs.* <400 ng/dl	1.55 (1.28–1.88)	**<0.001**	1.43 (1.15–1.77)	**0.001**
Platelets, per G/L	1.00 (0.99–1.00)	0.300	—	—

*P* values in bold denote statistical significance.

AFP, alpha-fetoprotein; ALBI, albumin-bilirubin; ArLD, alcohol-related liver disease; ECOG-PS, Eastern Cooperative Oncology Group performance status; MASLD metabolic dysfunction-associated steatotic liver disease.

**Fig. 3:**
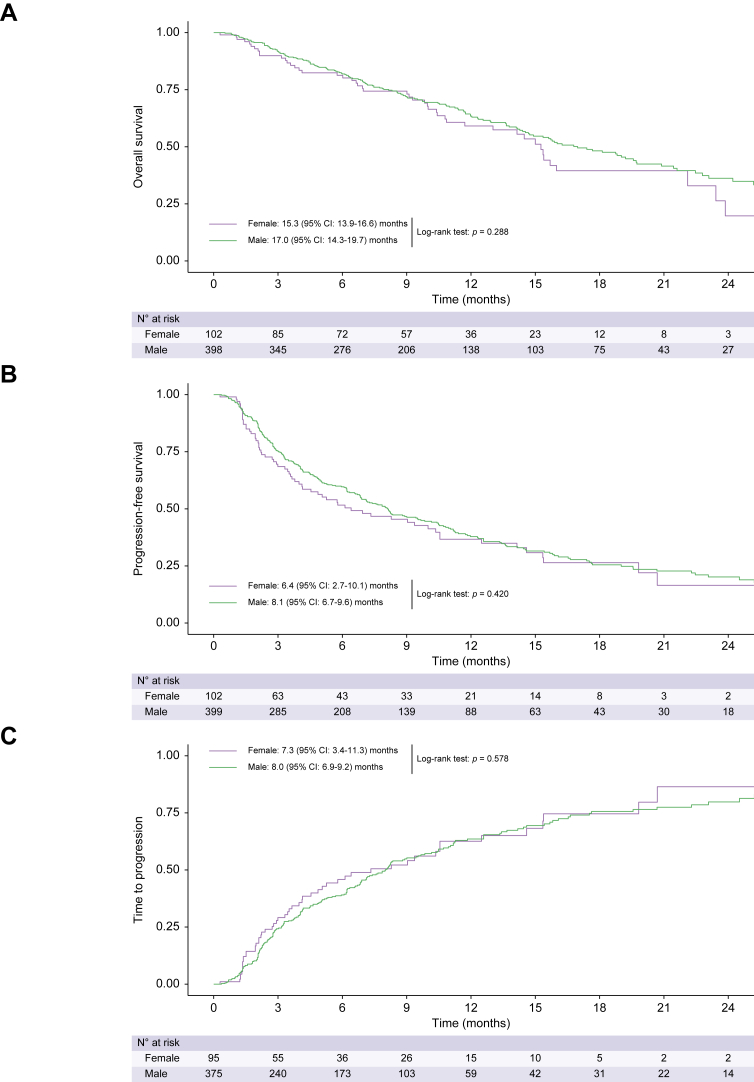
**Kaplan-Meier curves for overall survival, progression-free survival, and time to progression of patients with hepatocellular carcinoma treated with atezolizumab plus bevacizumab who fulfilled the main inclusion criteria of the IMbrave150 phase III trial according to sex.** (A) Overall survival, (B) progression-free survival, and (C) time to progression of patients with hepatocellular carcinoma treated with atezolizumab plus bevacizumab who fulfilled the main inclusion criteria of the IMbrave150 phase III trial according to sex.

Efficacy results were comparable when only including patients according to the main inclusion criteria of the pivotal IMbrave150 phase III study (n = 505, 60%) (Fig. 3, Table S1).

### Adverse events according to sex

**Fig. 4:**
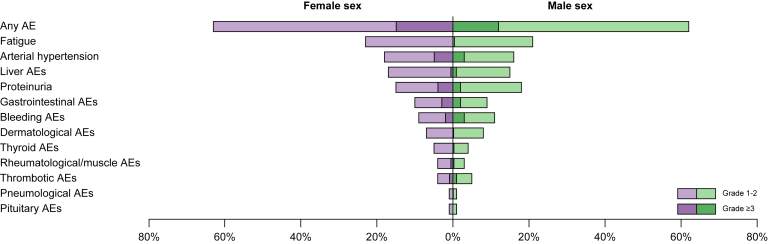
**AEs according to sex.** AEs, adverse events.

AEs are displayed in Table 4 and graphically depicted in Fig. 4. There were no significant differences in the rate, type, severity, and localisation of AEs between males and females (Table 4). Overall, the most common AEs included fatigue (females: n = 37, 23% *vs.* males: n = 143, 21%; *p =* 0.660), proteinuria (females: n = 25, 15% *vs.* males: n = 121, 18%; *p =* 0.443), and hypertension (females: n = 30, 18% *vs.* males: n = 109, 16%; *p =* 0.477). The most frequent high-grade (grade ≥3) AEs were hypertension (females: n = 8, 5% *vs.* males: n = 17, 3%), bleeding events (females: n = 3, 2% *vs.* males: n = 21, 3%), and proteinuria (females: n = 7, 4% *vs.* males: n = 16, 2%). Regarding immune-related adverse events, the number of low grade (*i.e*., grade 1/2; females: 36% *vs.* males: 30%; *p =* 0.245) and higher grade (*i.e*., grade 3–5; females: 4% *vs.* males: 4%; *p =* 0.764) events was similar between men and women.

**Table 4:** Comparison of treatment-emergent adverse events according to sex.

Follow-up characteristics	Study cohort, N = 840	Female, n = 163	Male, n = 677	*p* value
Any adverse event, n (%)	521 (62%)	103 (63%)	418 (62%)	0.733
Any severe adverse event, n (%)	104 (12%)	24 (15%)	80 (12%)	0.312
Any dermatological AE, n (%)	69 (8%)	12 (7%)	57 (8%)	0.659
Grade 1–2	68 (8%)	12 (7%)	56 (8%)	0.822
Grade 3–5	1 (0.1%)	—	1 (0.1%)	
Any gastrointestinal AE, n (%)	76 (9%)	16 (10%)	60 (9%)	0.703
Grade 1–2	58 (7%)	12 (7%)	46 (7%)	0.920
Grade 3–5	18 (2%)	4 (3%)	14 (2%)	
Any fatigue, n (%)	180 (21%)	37 (23%)	143 (21%)	0.660
Grade 1–2	177 (21%)	37 (23%)	140 (21%)	0.601
Grade 3–5	3 (0.4%)	—	3 (0.4%)	
Any liver AE, n (%)	130 (16%)	27 (17%)	103 (15%)	0.669
Grade 1–2	123 (15%)	26 (16%)	97 (14%)	0.826
Grade 3–5	7 (0.8%)	1 (0.6%)	6 (0.9%)	
Any thyroid AE, n (%)	37 (4%)	8 (5%)	29 (4%)	0.727
Grade 1–2	35 (4%)	8 (5%)	27 (4%)	0.686
Grade 3–5	2 (0.2%)	—	2 (0.3%)	
Any pituitary AE, n (%)	5 (0.6%)	—	5 (0.7%)	0.589
Grade 1–2	5 (0.6%)	—	5 (0.7%)	0.589
Any rheumatological/muscle AE, n (%)	24 (3%)	7 (4%)	17 (3%)	0.290
Grade 1–2	21 (3%)	6 (4%)	15 (2%)	0.462
Grade 3–5	3 (0.4%)	1 (0.6%)	2 (0.3%)	
Any pneumological AE, n (%)	6 (0.7%)	1 (0.6%)	5 (0.7%)	1.000
Grade 1–2	5 (0.6%)	1 (0.6%)	4 (0.6%)	0.886
Grade 3–5	1 (0.1%)	—	1 (0.1%)	
Any arterial hypertension, n (%)	139 (17%)	30 (18%)	109 (16%)	0.477
Grade 1–2	114 (14%)	22 (14%)	92 (14%)	0.270
Grade 3–5	25 (3%)	8 (5%)	17 (3%)	
Any proteinuria, n (%)	146 (17%)	25 (15%)	121 (18%)	0.443
Grade 1–2	123 (15%)	18 (11%)	105 (16%)	0.159
Grade 3–5	23 (3%)	7 (4%)	16 (2%)	
Any bleeding AE, n (%)	91 (11%)	14 (9%)	77 (11%)	0.304
Grade 1–2	67 (8%)	11 (7%)	56 (8%)	0.541
Grade 3–5	24 (3%)	3 (2%)	21 (3%)	
Any thrombotic AE, n (%)	38 (5%)	7 (4%)	31 (5%)	0.875
Grade 1–2	29 (4%)	5 (3%)	24 (4%)	0.935
Grade 3–5	9 (1%)	2 (1%)	7 (1%)	

Categorical variables were reported as absolute (n) and relative frequencies (%). Group comparisons of categorical variables were performed using either Chi-squared or Fisher’s exact test, as appropriate.

AE, adverse event.

## Discussion

Sex-related differences are known to affect innate and adaptive immune responses through a number of mechanisms including direct action of sex hormones on immune cell function, differential expression of genes located in sex chromosomes and altered epigenetic regulation of autosomal genetic material between the sexes. Sex-related diversity of nutritional status, gut microbial composition and disease-specific risk factors confer differential susceptibility to infection, autoimmunity, and response to vaccination.^7^

Whether efficacy and safety of immunotherapy might be different between the sexes is the matter of contention. A meta-analysis of 20 RCTs of ICI treatment in advanced or metastatic cancers found improved OS with immunotherapy in male and female patients affected mainly by advanced melanoma and lung cancer, with enhanced magnitude of benefit shown in males.^4^ In contrast, an updated meta-analysis of 23 RCTs including 13,721 trial participants challenged this finding and reported similar OS across the sexes.^5^ None of the published studies included patients with advanced HCC, where sex-related differences have been shown to influence the pathogenesis and progression of cirrhosis and cancer.^6^

In our meta-analysis of five eligible phase III RCTs in advanced HCC, a statistically significant OS benefit for immunotherapy *vs.* control arm was demonstrated in the male subgroup (pooled HR 0.79, 95% CI 0.73–0.86), but not in female patients (pooled HR 0.85, 0.70–1.03). This is interesting, as female patients are commonly underrepresented in phase III trials evaluating novel systemic therapies in patients with HCC, but results are commonly extrapolated to both sexes and current guidelines do not differ between male and female patients.^1^ Importantly, when directly comparing model estimates of male and female patients, no differences in the treatment effect between sexes were observed. The RATIONALE-301 was the only trial where the mortality risk was significantly reduced by immunotherapy in female patients but not in males. The reasons for this observation are unclear, especially since this was not observed in the KEYNOTE-240 trial with pembrolizumab, which is also an anti-PD-1 antibody. Subgroups of phase III trials are not balanced for other prognostic factors; thus, it could well be that negative prognostic factors (*e.g*., macrovascular invasion, metastasis, high AFP etc.) were more common in the male subgroup. However, since baseline characteristics for males and females are not provided separately in the RATIONALE-301 or any of the other phase III trials included, this remains only speculative.

Next, we compared the efficacy and safety of A+B directly between male and female patients with HCC using AB-Real, the largest and most geographically heterogeneous study of patients treated with A+B in routine practice. The large number of female patients accrued to AB-Real (n = 163), which is 2–3 times higher than in ICI arms of phase III trials in advanced HCC,^2^^,^[20], [21], [22], [23], [24], [25], [26], [27], [28] allowed for robust analyses, complemented by thorough appraisal of prognostically relevant subgroups across sexes. In the AB-Real study, neither univariable nor multivariable models adjusted for other relevant prognostic factors revealed any differences in OS, PFS, and TTP between females and males. Ancillary measures of efficacy including ORR and DCR were also equal across groups.

These results are in line with smaller retrospective studies of patients with HCC treated with A+B that reported no significant differences in OS and PFS between female and male patients.[29], [30], [31] Together, these findings do not suggest a significant association of patient sex with the efficacy of A+B in advanced HCC. However, it remains to be determined if this is specific to A+B or applies to other ICI mono- or combination therapies as well. Moreover, given the lack of a control group, we cannot appreciate whether the survival benefit derived from A+B might have been higher or lower than an alternative systemic therapy (*i.e*., TKI).

Autoimmune diseases are much more common in females,^32^^,^^33^ thus one could speculate that female individuals undergoing immunotherapy are at higher risk of developing immune-related AEs. Indeed, in a recently published paper including an FDA-pooled analysis of landmark trials in HCC and a multi-institutional dataset including over 357 patients with HCC treated with ICIs, the relative emergence of treatment-related AEs of grade ≥2 was higher in females.^34^ In contrast, we did not observe any differences in type, location, severity, or frequency of AEs in female compared to male patients. Notably, the FDA data analysis and multi-institutional cohort did not include patients treated with A+B, and the observed differences might have been driven by other treatments (*e.g*., anti-cytotoxic T-lymphocyte-associated protein 4).^34^

The meta-analysis has several limitations. Firstly, it was not based on individual participant data and subgroups were not stratified for other relevant prognostic factors, which could lead to imbalances between male and female subgroups and treatment arms. Secondly, several phase III RCTs could not be included as they did not report sub-analyses of OS stratified by sex. Thirdly, the subgroups are not balanced, with the sample size being larger in males, and the number of female patients in each trial being small. Finally, the phase III RCTs included were heterogeneous in terms of control arm and line of treatment. Therefore, the results of this meta-analysis can only be considered hypothesis-generating.

The real-world study has also some limitations beyond the well-known shortcomings of retrospective studies. The lack of a control group prevents conclusions on a potential higher relative benefit from immunotherapy *vs*. an alternative systemic therapy in females or males. The imaging schedule as well as modality was not pre-specified due to the real-world nature of the study. Since sex was based on self-reporting, it is possible that a very small proportion of included individuals might not be 46XX or 46XY; however, we assume that this would not have had a relevant impact on our results.^18^

In conclusion, while a slightly lower efficacy of immunotherapy in female patients with HCC was suggested in a meta-analysis of the sex-specific HRs for OS of five phase III RCTs, this could neither be confirmed when directly assessing the treatment effect differences between sexes in a similar meta-analysis, nor in a large global real-world cohort of patients treated with A+B, where efficacy and safety between males and females were similar.

Whilst treatment allocation based on patient sex is not recommended, our findings warrant continued investigation of sex-related differences as a determinant of responsiveness to ICIs in patients with advanced HCC.

## Financial support

No financial support specific to this study was received.

## Authors’ contributions

Concept of the study (L.B., B.Sc., D.J.P., M.P.), data collection (all authors), statistical analysis (L.B., M.P.), drafting of the manuscript (L.B., B.Sc., D.J.P., M.P.), revision for important intellectual content and approval of the final manuscript (all authors).

## Data availability statement

The data that support the findings of this study are available from the corresponding author upon reasonable request.

## Conflicts of interest

The authors have nothing to disclose regarding the work under consideration for publication. The following authors disclose conflicts of interests outside the submitted work: L.B. has nothing to disclose. B.Sc. received travel support from AbbVie, AstraZeneca, Gilead and Ipsen as well as grant support from AstraZeneca. C.A.M.F. has nothing to disclose. A.D. is supported by the National Institute for Health Research (NIHR) Imperial BRC, by grant funding from the European Association for the Study of the Liver (2021 Andrew Burroughs Fellowship) and from Cancer Research UK (RCCPDB- Nov21/100008). A.D. received educational support for congress attendance and consultancy fees from Roche. K.P. has nothing to disclose. M.B.R. has nothing to disclose. E.L.M. is a salaried employee of Berry Consultants. J.C. has nothing to disclose. N.N. has nothing to disclose. P.-C.L. has nothing to disclose. L.W. has nothing to disclose. C.A. has nothing to disclose. A.K. has nothing to disclose. An.S. has nothing to disclose. B.St. has nothing to disclose. A.Ca. has nothing to disclose. T.P. has nothing to disclose. Y.I.A. has nothing to disclose. S.C. has nothing to disclose. B.W. has nothing to disclose. A.P. has nothing to disclose. Y.-H.H. has nothing to disclose. S.P. has nothing to disclose. C.V. has nothing to disclose. F.S. has nothing to disclose. G.M. has nothing to disclose. D.B. has nothing to disclose. A.V. has nothing to disclose. J.v.F. has received advisory board fees from Roche. K.S. has nothing to disclose. M.S. has nothing to disclose. M.T. served as a speaker and/or consultant and/or advisory board member for Albireo, BiomX, Falk, Boehringer Ingelheim, Bristol-Myers Squibb, Falk, Genfit, Gilead, Hightide, Intercept, Janssen, MSD, Novartis, Phenex, Pliant, Regulus, Siemens and Shire, and received travel support from AbbVie, Falk, Gilead, and Intercept as well as grants/research support from Albireo, Alnylam, Cymabay, Falk, Gilead, Intercept, MSD, Takeda, and UltraGenyx. He is also co-inventor of patents on the medical use of 24-norursodeoxycholic acid. Ad.S. is supported by grant funding from CRUK, served as a speaker for Merck and Chugai and received grants from Histosonics, Transgene, Oncolytics and Theolytics. H.W. has received lecture and consulting fees from AstraZeneca, Roche, and Eisai. F.P. has received honoraria for advisory board or lecturing from Astrazeneca, Bayer, Bracco, ESAOTE, EISAI, Exact Sciences, GE, IPSEN, MSD, Roche, Samsung, Siemens Healthineers. P.R.G. received honoraria from Bayer, Boston Scientific, AstraZeneca, Adaptimmune, BMS, Eisai, MSD, Sirtex, Lilly, Roche, Guerbet, Ipsen and Daiichi-Sankyo. R.S. has nothing to disclose. M.K. received lecture fees from Eli Lilly, Bayer, Eisai, Chugai, Takeda, AstraZeneca as well as grant support from Taiho, Otsuka, EA Pharma, AbbVie, Eisai, Chugai, GE Healthcare; and acts on advisory boards from Chugai, Roche, AstraZeneca, Eisai. A.G.S. has served as a consultant or on advisory boards for Genentech, AztraZeneca, Eisai, Exelixis, Bayer, Boston Scientific, FujiFilm Medical Sciences, Exact Sciences, Roche, Glycotest, Freenome, and GRAIL. Dr. Singal’s research is conducted with support from National Cancer Institute R01 MD012565 and R01 CA256977. A.I. has nothing to disclose. S.V.U. has served on advisory boards for Eisai, Astra Zeneca, IgM biosciences and received institutional support for research from AbbVie, Inc, Adlai Nortye, ArQule, Inc, AstraZeneca, Atreca, Boehringer Ingelheim, Bristol-Myers Squibb, Celgene Corporation, Ciclomed LLC, Erasca, Evelo Biosciences, Inc, Exelexis, G1 Therapeutics, Inc, GlaxoSmithKline GSK, IGM biosciences, Incyte, Isofol, Klus Pharma, Inc, Macrogenics, Merck Co. Inc, Mersana Therapeutics, OncoMed Pharmaceuticals, Inc, Pfizer, Regeneron, Inc, Revolution Medicines, Inc, Synermore Biologics Co, Takeda, Tarveda Therapeutics, Tesaro, Tempest, Vigeo Therapeutics Inc. (all funds to institution). N.D.P. serves as a consultant for Exact Sciences, Eli Lilly, Freenome, Astra Zeneca and has served on advisory boards of Genentech, Eisai, Bayer, Exelixis, Wako/Fujifilm and has received research funding from Bayer, Target Pharmasolutions, Exact Sciences, and Glycotest. A.Co. served as consultant/advisory role for AstraZeneca, BMS, MSD, Roche, IQVIA and OncoC4. He also received speaker’s fees from AstraZeneca, Pierre-Fabre, EISAI. A.K. has nothing to disclose. L.R. reports consulting fees from AstraZeneca, Basilea, Bayer, BMS, Eisai, Exelixis, Genenta, Hengrui, Incyte, Ipsen, IQVIA, Lilly, MSD, Nerviano Medical Sciences, Roche, Servier, Taiho Oncology, Zymeworks; lecture fees from AstraZeneca, Bayer, Eisai, Gilead, Incyte, Ipsen, Merck, Serono, Roche, Sanofi, Servier; travel expenses from AstraZeneca; research grants (to Institution) from Agios, AstraZeneca, BeiGene, Eisai, Exelixis, Fibrogen, Incyte, Ipsen, Lilly, MSD, Nerviano Medical Sciences, Roche, Zymeworks. H.J.C. has nothing to disclose. D.J.P. is supported by grant funding from the Wellcome Trust Strategic Fund (PS3416) and acknowledges grant support from the Cancer Treatment and Research Trust (CTRT); the NIHR Imperial Biomedical Research Centre; and the AIRC MFAG Grant No. 25697, Associazione Italiana per la Ricerca sul Cancro Foundation, Milan, Italy. D.J.P. acknowledges the following COIs: Lecture fees: Bayer Healthcare, Astra Zeneca, EISAI, Bristol-Myers-Squibb, Roche, Ipsen; Travel expenses: Bristol-Myers-Squibb, Roche, Bayer Healthcare; Consulting fees: Mina Therapeutics, Boeringer Ingelheim, Ewopharma, EISAI, Ipsen, Roche, H3B, Astra Zeneca, DaVolterra, Mursla, Avammune Therapeutics, LiFT Biosciences, Exact Sciences; Research funding (to institution): MSD, BMS, GSK. M.P. served as a speaker and/or consultant and/or advisory board member for Astra Zeneca, Bayer, Bristol-Myers Squibb, Eisai, Ipsen, Lilly, MSD, and Roche, and received travel support from Bayer, Bristol-Myers Squibb, Ipsen, and Roche.

Please refer to the accompanying ICMJE disclosure forms for further details.
