# Supplementary material for: A meta-analysis and real-world cohort study on the sex-related differences in efficacy and safety of immunotherapy for hepatocellular carcinoma
Source: JHEP Rep. 2023 Dec 12;6(2):100982. doi: 10.1016/j.jhepr.2023.100982 (PMC10809085; doi:10.1016/j.jhepr.2023.100982)
Supplement: Multimedia component 1 [file mmc1.pdf]

A meta-analysis and real-world cohort study on the sex-related differences in efficacy and safety of immunotherapy for hepatocellular carcinoma

Lorenz Balcar, Bernhard Scheiner, Claudia Angela Maria Fulgenzi, Antonio D'Alessio, Katharina Pomej, Marta Bofill Roig, Elias Laurin Meyer, Jaekyung Che, Naoshi Nishida, Pei-Chang Lee, Linda Wu, Celina Ang, Anja Krall, Anwaar Saeed, Bernardo Stefanini, Antonella Cammarota, Tiziana Pressiani, Yehia I. Abugabal, Shadi Chamseddine, Brooke Wietharn, Alessandro Parisi, Yi-Hsiang Huang, Samuel Phen, Caterina Vivaldi, Francesca Salani, Gianluca Masi, Dominik Bettinger, Arndt Vogel, Johann von Felden, Kornelius Schulze, Marianna Silletta, Michael Trauner, Adel Samson, Henning Wege, Fabio Piscaglia, Peter R. Galle, Rudolf Stauber, Masatoshi Kudo, Amit G. Singal, Aleena Itani, Susanna V. Ulahannan, Neehar D. Parikh, Alessio Cortellini, Ahmed Kaseb, Lorenza Rimassa, Hong Jae Chon, David J. Pinato, Matthias Pinter

Table of contents

Fig. S1 2

Fig. S2 3

Fig. S3 4

Fig. S4 5

Supplementary Methods 1 6

Table S1 8

Table S2 9

Table S3 10

Fig. S1

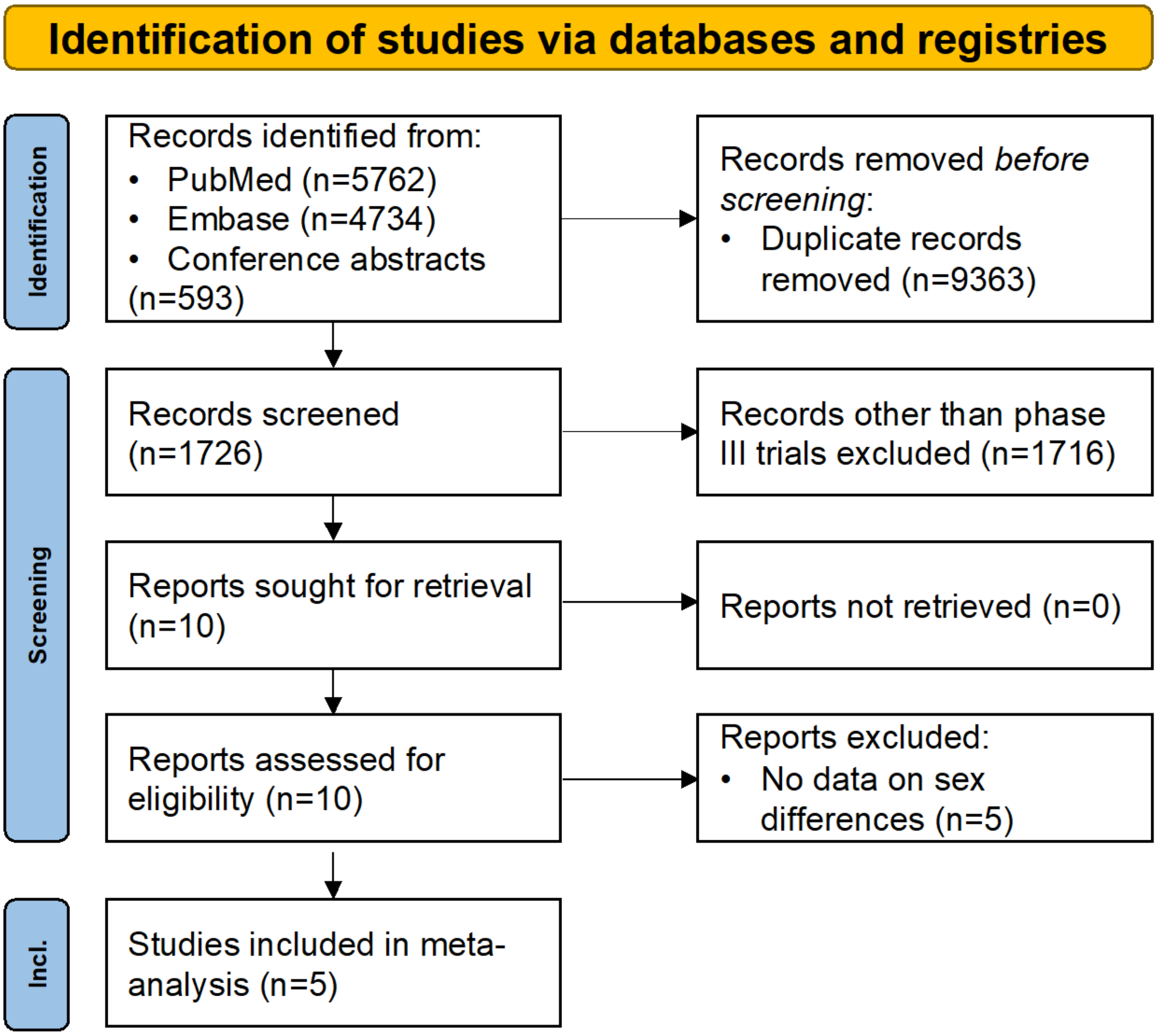

Fig. S1. PRISMA chart reporting the results of the research strategy

Fig. S2

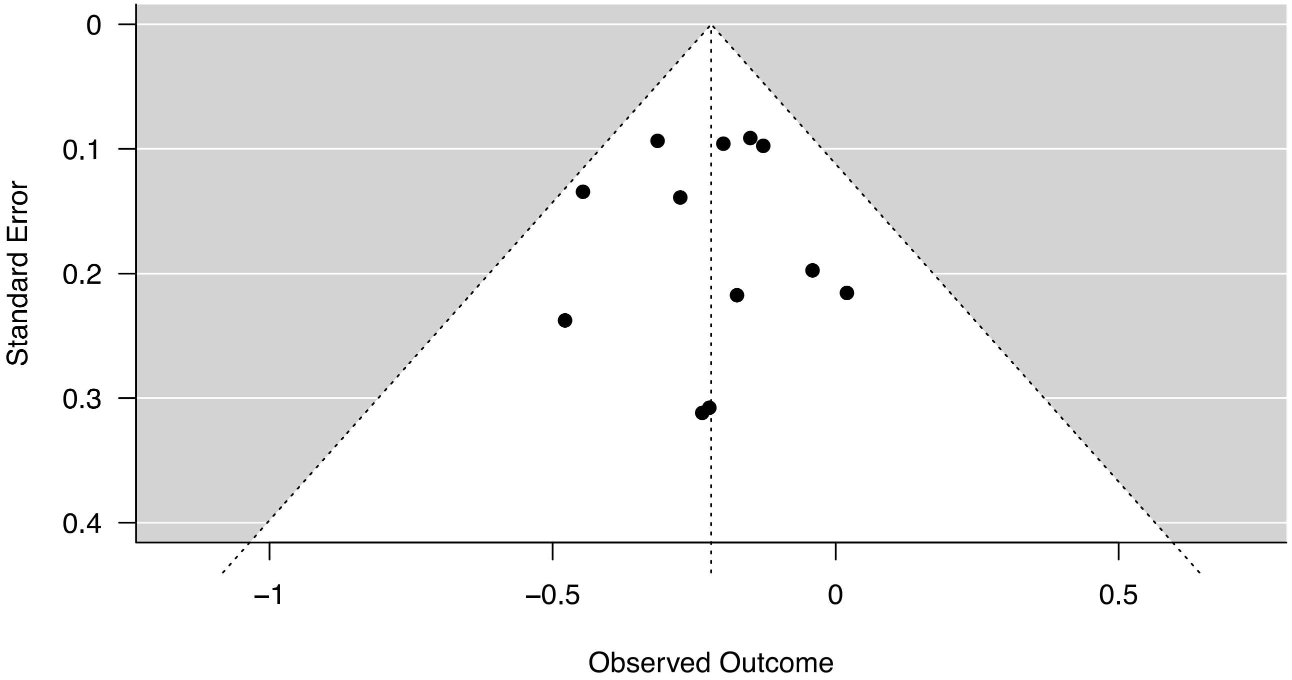

Fig. S2. Funnel-plot displaying each study included in the meta-analysis according to sex, indicating low inclusion bias of included studies. Each dot represents a single study. The y-axis shows the standard error of the effect estimate, while the x-axis shows the observed outcomes for the respective studies. The outer dashed lines indicate the triangular region within which 95% of studies are expected to lie in the absence of both biases and heterogeneity. The dashed vertical line corresponds to no intervention effect

Fig. S3

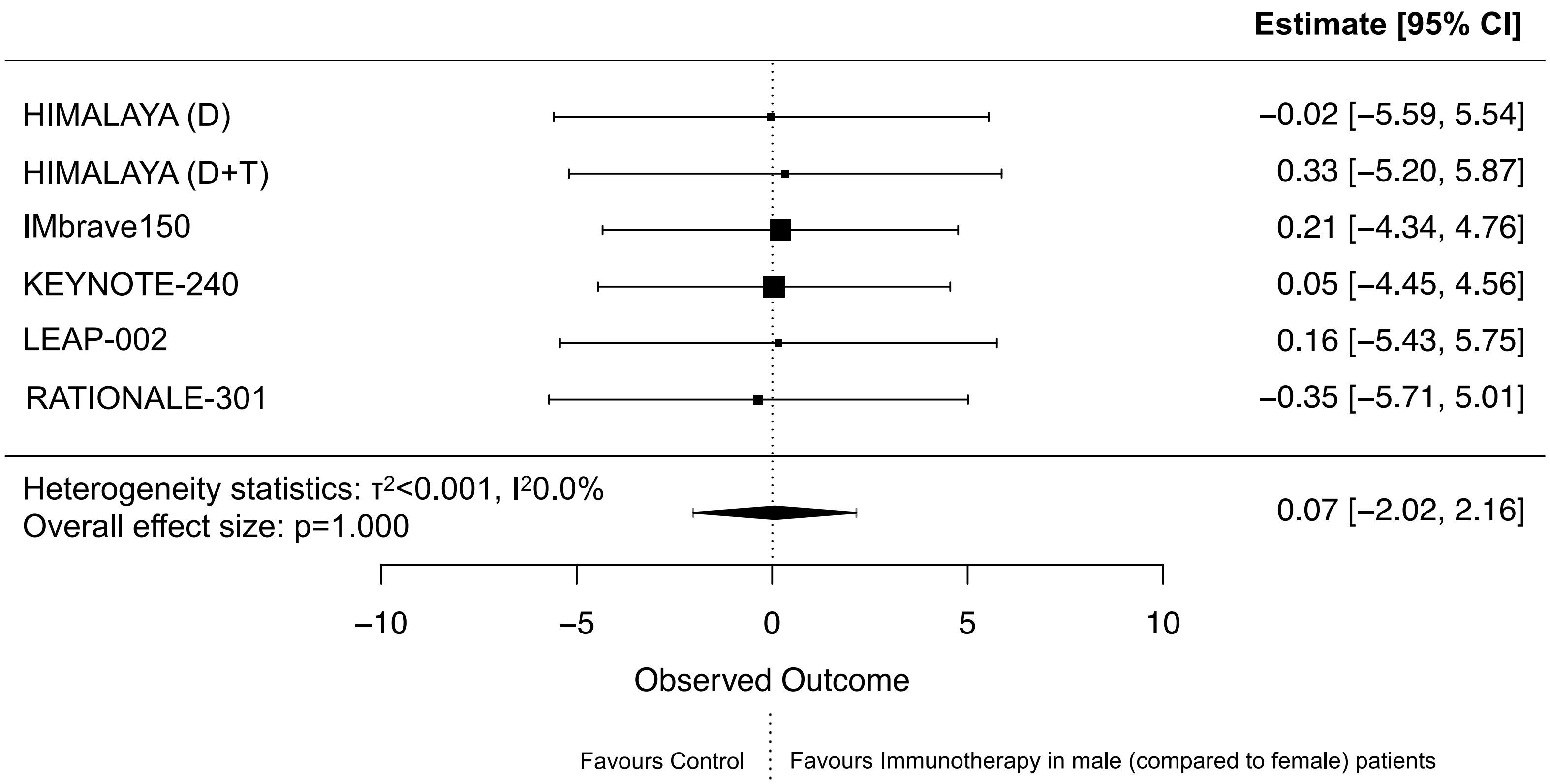

Fig. S3. Forrest plot of a random effect meta-analysis of the differences between male and female coefficients of the Cox model of the phase III trials. Positive values correspond to a greater effect of immunotherapy in male compared to female patients

Fig. S4

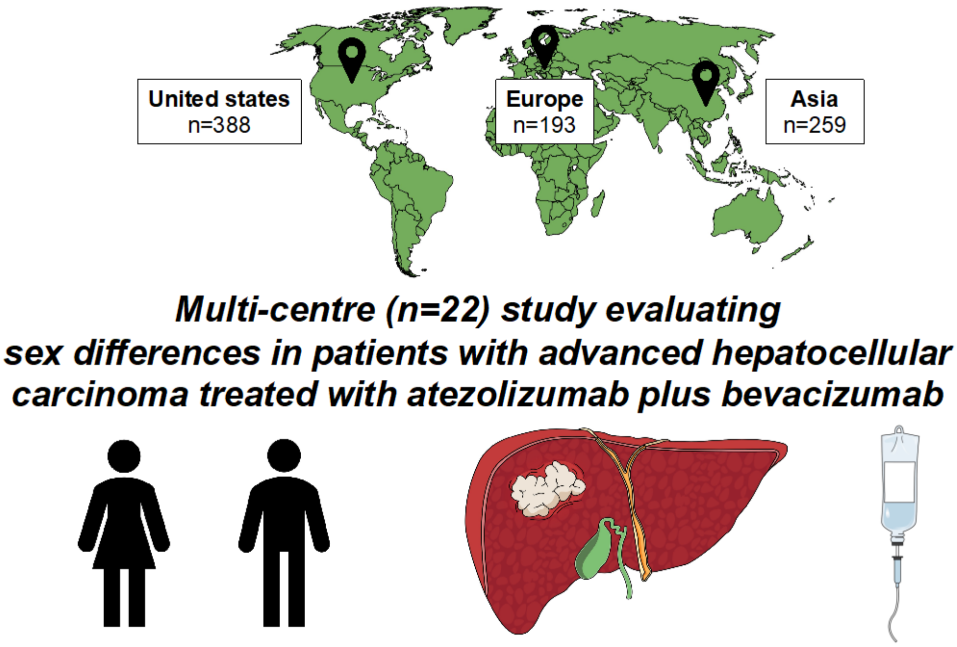

Fig. S4. Study description

Supplementary Methods 1. Search terms applied to publication databases and secondary sources.

Filters applied: Clinical Trial, Phase III, from 2007/1/1 - 2023/5/21

1. exp Carcinoma, Hepatocellular/ or exp Liver Neoplasms/

2. HCC

3. (hepat* or liver) adj3 (neoplasm* or cancer* or tumor* or malignan* or carcinoma*)

4. 1 or 2 or 3

5. Exp Atezolizumab/

6. (atezolizumab or tecentriq* or mpdl 3280* or mpdl3280* or rg 7446 or rg7446)

7. exp Nivolumab/

8. (nivolumab or opdivo* or bms-936558 or mdx-1106 or ono-4538 or bms936558 or 'mdx1106' or 'ono4538')

9. exp Bevacizumab/

10. (bevacizumab or avastin*)

11. exp Durvalumab/

12. (durvalumab or imfinzi* or medi4736 or medi-4736)

13. exp Tremelimumab/

14. (tremelimumab or cp-675206*)

15. exp Sintilimab/

16. (sintilimab or tyvyt* or ibi308*)

17. exp IBI305/

18. exp Donafenib/

19. (donafenib or cm 4307 or donafenib tosilate or donafenib tosylate or zeprosen* or zeprosyn*)

20. exp Pembrolizumab/

21. pembrolizumab

22. exp Tislelizumab/

23. (tislelizumab or BGB-A317)

24. exp Camrelizumab/

25. (camrelizumab or AiRuiKa)

26. exp Rivoceranib/

27. (rivoceranib or apatinib)

28. exp Sunitinib/

29. (sunitinib or Sutent or SU11248)

30. exp Brivanib/

31. (Brivanib or Brivanib alaninate or BMS582664)

32. exp Linifanib/

33. (linifanib or ABT869)

34. 5 or 6 or 7 or 8 or 9 or 10 or 11 or 12 or 13 or 14 or 15 or 16 or 17 or 18 or 19 or 20 or 21 or 22 or 23 or 24 or 25 or 26 or 27 or 28 or 29 or 30 or 31 or 32 or 33 or 34 or 35 or 36

Table S1

Name	Inclusion criteria	Experimental arm	Control arm	Primary endpoint(s)	Secondary endpoint(s)	Sample size
**IMbrave150**	Advanced HCC 1st line CTP A ECOG 0-1, treated varices	Atezolizumab + Bevacizumab	Sorafenib	OS and PFS (coprimary)	ORR DOR QoL	336 (A+B) 165 (S)
**HIMALAYA**	Advanced HCC 1st line CTPA ECOG 0-1 no MVI at vp4	Durvalumab + Tremelimumab<br><br>Durvalumab	Sorafenib	OS of D+T vs. S	OS for D vs. S (non-inferiority) ORR for D+T and D alone PFS DOR DCR	1324 (total) 393 (D+T) 389 (D) 389 (S)
**KEYNOTE-240**	Advanced HCC 2nd line CTPA ECOG 0-1 no MVI at vp4	Pembrolizumab	Placebo	OS and PFS (coprimary)	ORR DOR TTP Safety Tolerability	278 (Pe) 135 (Pl)
**LEAP-002**	Advanced HCC 1st line CTPA ECOG 0-1, no MVI at vp4 or bile duct invasion	Lenvatinib + Pembrolizumab	Lenvatinib + Placebo	OS and PFS (coprimary)	ORR DOR Safety Tolerability	395 (L+Pe) 399 (L+Pl)
**RATIONALE-301**	Advanced HCC 1st line CTPA ECOG 0 or 1, no thrombus MVI at vp4 or IVC	Tislelizumab	Sorafenib	OS (non-inferiority)	ORR PFS DOR Safety	342 (T) 332 (S)

Table S1. Description of the trials included in the meta-analysis

Table S2

Name	Random sequence generation	Allocation concealment	Blinding of participants and personnel	Blinding of outcome assessment	Incomplete outcome data	Selective reporting	Other bias
**IMbrave150**	Low risk	Low risk	High risk	Low risk	Low risk	Low risk	Low risk
**HIMALAYA**	Low risk	Low risk	High risk	High risk	Low risk	Low risk	Low risk
**KEYNOTE-240**	Low risk	Low risk	Low risk	Low risk	Low risk	Low risk	Low risk
**LEAP-002**	Low risk	Low risk	Low risk	Low risk	Low risk	Low risk	Low risk
**RATIONALE-301**	Low risk	Low risk	Low risk	Low risk	Low risk	Low risk	Low risk

Table S2. Risk of bias assessment according to the Cochrane risk of bias assessment tool

Table S3

Overall survival	**Univariable**		**Multivariable**	
	**HR (95%CI)**	**p-value**	**aHR (95%CI)**	**p-value**
Age, per year	1.00 (0.99-1.01)	0.748	-	-
Sex, male vs. female	0.84 (0.61-1.16)	0.289	0.73 (0.52-1.03)	0.073
Cirrhosis, vs. non-cirrhotic	0.98 (0.72-1.34)	0.904	-	-
Aetiology				
ArLD	1	-	-	-
Viral	0.99 (0.66-1.49)	0.965	-	-
MASLD	1.54 (0.91-2.59)	0.107	-	-
ArLD/Viral	1.34 (0.82-2.19)	0.244	-	-
Other/unknown	1.01 (0.60-1.72)	0.976	-	-
Presence of ascites	1.32 (0.92-1.89)	0.138	-	-
ALBI score, per point	1.52 (1.21-1.90)	**<0.001**	2.70 (2.03-3.58)	**<0.001**
Macrovascular invasion	1.65 (1.23-2.21)	**<0.001**	1.53 (1.14-2.06)	**0.005**
Extrahepatic spread	0.93 (0.71-1.22)	0.604	-	-
ECOG PS ≥1, vs. 0	1.13 (0.86-1.48)	0.386	-	-
AFP, ≥400 ng/dL vs. <400 ng/dL	1.45 (1.11-1.90)	**0.007**	1.24 (0.93-1.66)	0.143
Platelets, per G/L	1.00 (0.99-1.00)	0.548	-	-
Progression-free survival	**Univariable**		**Multivariable**	
	**HR (95%CI)**	**p-value**	**aHR (95%CI)**	**p-value**
Age, per year	1.00 (0.99-1.01)	0.781	-	-
Sex, male vs. female	0.89 (0.68-1.18)	0.420	0.89 (0.67-1.20)	0.460
Cirrhosis, vs. non-cirrhotic	0.97 (0.75-1.25)	0.824	-	-
Aetiology				
ArLD	1	-	-	-
Viral	1.18 (0.84-1.64)	0.344	-	-
MASLD	1.38 (0.89-2.14)	0.151	-	-
ArLD/Viral	1.24 (0.80-1.90)	0.339	-	-
Other/unknown	1.03 (0.66-1.59)	0.911	-	-
Presence of ascites	1.37 (1.02-1.83)	**0.035**	1.27 (0.93-1.74)	0.139
ALBI score, per point	1.66 (1.44-1.91)	**<0.001**	1.44 (1.14-1.83)	**0.002**
Macrovascular invasion	1.34 (1.05-1.71)	**0.021**	1.26 (0.98-1.62)	0.074
Extrahepatic spread	1.11 (0.89-1.39)	0.345	-	-
ECOG PS ≥1, vs. 0	1.37 (1.10-1.71)	**0.006**	1.41 (1.12-1.80)	**0.004**
AFP, ≥400 ng/dL vs. <400 ng/dL	1.36 (1.09-1.71)	**0.007**	1.28 (1.01-1.64)	**0.046**
Platelets, per G/L	1.00 (0.99-1.00)	0.823	-	-
Time to progression	**Univariable**		**Multivariable**	
	**HR (95%CI)**	**p-value**	**aHR (95%CI)**	**p-value**
Age, per year	1.00 (0.99-1.01)	0.746	-	-
Sex, male vs. female	0.92 (0.68-1.25)	0.588	0.91 (0.67-1.24)	0.539
Cirrhosis, vs. non-cirrhotic	0.85 (0.65-1.11)	0.231	-	-

Aetiology				
ArLD				
Viral	1.26 (0.86-1.87)	0.239	-	-
MASLD	1.43 (0.86-2.37)	0.169	-	-
ArLD/Viral	1.35 (0.83-2.21)	0.226	-	-
Other/unknown	1.23 (0.75-2.02)	0.408	-	-
Presence of ascites	1.18 (0.86-1.63)	0.304	-	-
ALBI score, per point	1.30 (1.02-1.67)	**0.037**	1.27 (0.99-1.63)	0.065
Macrovascular invasion	1.24 (0.95-1.63)	0.114	-	-
Extrahepatic spread	1.06 (0.83-1.35)	0.626	-	-
ECOG PS ≥1, vs. 0	1.30 (1.02-1.66)	**0.036**	1.33 (1.04-1.70)	**0.025**
AFP, ≥400 ng/dL vs. <400 ng/dL	1.35 (1.05-1.73)	**0.019**	1.33 (1.03-1.71)	**0.026**
Platelets, per G/L	1.00 (0.99-1.00)	0.998	-	-

Table S3. Uni- and multivariable Cox regression analyses of factors associated with overall survival, progression-free survival, and time to progression in patients with hepatocellular carcinoma treated with atezolizumab plus bevacizumab who fulfilled the main inclusion criteria of the IMbrave150 phase III trial.

Abbreviations: AFP alpha-fetoprotein; ALBI albumin-to-bilirubin; ArLD alcohol-related liver disease; ECOG PS Eastern Cooperative Oncology Group Performance Status; MASLD metabolic-associated steatotic liver disease
